# Direction-of-Arrival Estimation in Coprime Array Using the ESPRIT-Based Method

**DOI:** 10.3390/s19030707

**Published:** 2019-02-09

**Authors:** Zhen Meng, Weidong Zhou

**Affiliations:** College of Automation, Harbin Engineering University, Harbin 150001, China; mengzhenheu@163.com

**Keywords:** direction-of-arrival estimation, degrees-of-freedom, coprime array, Toeplitz covariance matrix, virtual uniform linear array, ESPRIT

## Abstract

Coprime arrays have shown potential advantages for direction-of-arrival (DOA) estimation by increasing the number of degrees-of-freedom in the difference coarray domain with fewer physical sensors. In this paper, a new DOA estimation algorithm for coprime array based on the estimation of signal parameter via rotational invariance techniques (ESPRIT) is proposed. We firstly derive the observation vector of the virtual uniform linear array but the covariance matrix of this observation vector is rank-deficient. Different from the traditional Toeplitz matrix reconstruction method using the observation vector, we propose a modified Toeplitz matrix reconstruction method using any non-zero row of the covariance matrix in the virtual uniform linear array. It can be proved in theory that the reconstructed Toeplitz covariance matrix has full rank. Therefore, the improved ESPRIT method can be used for DOA estimation without peak searching. Finally, the closed-form solution for DOA estimation in coprime array is obtained. Compared to the traditional coprime multiple signal classification (MUSIC) methods, the proposed method circumvents the use of spatial smoothing technique, which usually results in performance degradation and heavy computational burden. The effectiveness of the proposed method is demonstrated by numerical examples.

## 1. Introduction

Direction-of-arrival (DOA) estimation plays a vital role in radar, sonar, wireless communications, remote sensing and other engineering applications [1,2,3,4,5]. Specially, accurate DOA estimation is important for monitoring the electromagnetic environment where the signals captured from radars are usually distorted depending on weather condition. To measure these signal parameters quickly and precisely, certain techniques combined with artificial neural networks [6,7] and target identification algorithm [8] have been developed. A large number of DOA estimation methods have been proposed in the past decades. Multiple signal classification (MUSIC) [9], estimation of signal parameters via rotational invariance techniques (ESPRIT) [10] and their variants [11,12,13,14] are the popular subspace methods for DOA estimation, which can provide higher resolution performance than the traditional beamforming [15,16,17,18] and time difference estimation algorithms [19]. It is well known that these methods can detect up to *N* − 1 sources in a uniform linear array (ULA) with *N* sensors. If more sources have to be detected, these methods require more array sensors. As a result, the hardware cost and the computational burden will be increased significantly. In other words, for the traditional DOA estimation methods focusing on the ULA geometry, the available degrees-of-freedom (DOFs) are restricted by the number of array sensors.

Sparse arrays can increase the number of DOFs, which have attracted the attention of many researchers. Minimum redundancy array [20] and minimum hole array [21] are the two examples of sparse arrays. In the coarray domain, minimum redundancy array can maximize the number of virtual sensors, while minimum hole array can minimize the number of holes. However, the actual positions of sensors in these two sparse arrays cannot be obtained in analytical form. The nested arrays [22,23,24] and coprime arrays [25] have recently been researched. Both array forms have spare geometries. However, there are mutual coupling effects in the nested arrays because some sensor elements are placed closely. A coprime array consists of a coprime pair of ULAs with inter-element spacing larger than half wavelength. Coprime arrays can achieve much more DOFs than the number of physical sensors, and there is no mutual coupling problem. Therefore, coprime arrays have triggered extensive research in array signal processing because of its potential advantages. In [26,27], the coprime sensor arrays are extended in beamforming to achieve peak side lobe attenuation. The general coprime sampling scheme is proposed in [28] to conduct efficient compression of Toeplitz covariance matrices for spatial spectrum estimation. In [29], the authors relate the coprime DFT filter banks in array signal processing to the interpolated finite impulse response filter design. The theory of coprime sampling has been introduced to multidimensional array design in [30]. Similarly, the advantages of coprime arrays can also be exploited for DOA estimation.

Some studies have been conducted by utilizing coprime arrays to provide super resolution for DOA estimation. By combining the estimated results of two decomposed linear subarrays based on the MUSIC method, the DOA estimation could be achieved for coprime array in [31]. However, the total spectral search step results in high computational complexity. In order to narrow the search sector, the authors in [32] propose a partial spectral search algorithm in one-dimensional coprime linear array. However, the estimation accuracy is not improved compared to the total spectral search algorithm. In [33], the partial spectral search algorithm has been applied in the two-dimensional coprime planar array. By using the linear relationship in transformed domain, the DOA of sources can be retrieved in a small limited sector. However, the high-dimensional computation still requires heavy computational burden. A fast search-free DOA estimation algorithm for coprime array has been developed in [34]. By projecting the estimated results in two-dimensional plane onto one-dimensional angle region, the DOA can be estimated by combining the estimated results of two subarrays. However, the methods mentioned above do not fully utilize the increased DOFs of the coprime array. In order to fully employ the DOFs contained in coprime array for DOA estimation, a novel method in [35] vectorizes the coprime array covariance matrix to formulate a larger virtual ULA with widened aperture. Taking the vectorized coprime array covariance matrix as an observation vector, the spatial smoothing technique is applied to construct a full-rank covariance matrix for the virtual ULA. Based on the smoothed covariance matrix, MUSIC algorithm is directly performed to identify more sources. However, the achieved aperture of the virtual ULA can be significantly reduced due to the implementation of the spatial smoothing technique, which results in a trade-off between the DOFs and the performance. Moreover, the computational complexity is greatly increased arising from the spatial smoothing and the spectral search steps. To use the sparsity of sources in the coprime array, the least absolute shrinkage and selection operator is exploited to formulate an optimization problem for detecting the DOAs of sources in [36]. However, the regularization variable is difficult to determine and a fixed number will lead to spurious estimations in some cases. In addition, the optimization problem has to be solved by using the optimization programming in high computational cost. Therefore, it is still an urgent task for us to improve the DOA estimation performance in coprime array with low computational complexity.

In this paper, we have proposed a novel DOA estimation algorithm in the coprime array by using the ESPRIT-based method. Since the observation vector of the virtual uniform linear array behaves like single snapshot, the rank of its covariance matrix needs to be restored. Unlike the traditional Toeplitz matrix reconstruction method [37] using the observation vector, we reconstruct a Toeplitz covariance matrix using any non-zero row of the covariance matrix in the virtual uniform linear array. Moreover, the symmetrical structure of the virtual uniform linear array is fully utilized for the reconstruction. Theoretically, we can prove that the rank of the reconstructed Toeplitz covariance matrix is full. Due to the avoidance of the spatial smoothing technique in the traditional coprime MUSIC methods, the available DOFs can be fully utilized and the computational complexity can be greatly reduced. Then the modified ESPRIT is adopted for DOA estimation to avoid the predefined spatial sampling grids and the spectrum searching step. Finally, the closed-form expression for DOA estimation is obtained. Because of increased DOF in the coprime coarray, the proposed method can detect more DOA than the ULA with the same number of sensors. Simulated results demonstrate that the proposed method has higher accuracy and lower computational cost than the existing coprime array DOA estimation methods. 

The remainder of the paper is arranged as follows. In Section 2, we present the coprime array configuration and the array signal model for DOA estimation. The proposed coprime array DOA estimation algorithm and the related remarks are described in Section 3. In Section 4, experimental results and the corresponding discussions are given to verify the effectiveness of the proposed algorithm. Finally, Section 5 concludes this paper.

## 2. Problem Formulation

**Figure 1:**
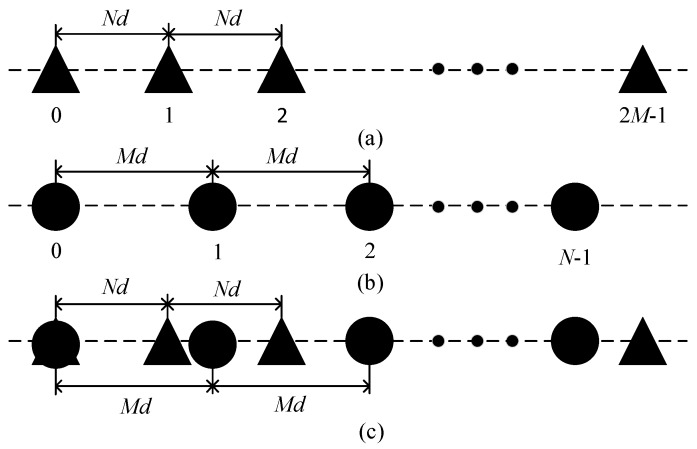
Illustration of coprime array configuration: (**a**) the first subarray; (**b**) the second subarray; (**c**) the generated coprime array.

Let us consider a coprime array consisting of two uniform linear subarrays, which is illustrated in Figure 1. The first subarray contains 2*M* physical sensors with *Nd* inter-element spacing, where *N* and *M* are coprime integers and *d* is set as half of signal wavelength, or λ/2. The other subarray has *N* sensors with *Md* inter-element spacing. The first sensor of these two subarrays can be collocated at the zeroth position for reference. Consequently, the resulted coprime array has *N* + 2*M* − 1 sensors in total. We assume that the positions of the coprime array sensors are located in the vector:

(1) $$k={\left[k_{1},k_{2},\cdots ,k_{N+2M-1}\right]}^{T}$$

where $k_{1}=0$ and $k_{i},i=1,2,\cdots ,N+2M-1$ belong to the following set

(2) S={Mnd,0≤n≤N−1}∪{Nmd,0≤m≤2M−1}

There are *P* uncorrelated narrowband signals impinging on the coprime array from the far filed with the directions:

(3) $$\Theta ={\left[\theta_{1},\theta_{2},\cdots ,\theta_{P}\right]}^{T}$$

where $\theta_{p}$ denotes the direction of the *p*-th signal. The received data vector in the coprime array at time index *t* is given by:

(4) $$x(t)=\sum_{p=1}^{P}a(\theta_{p})s_{p}(t)+n(t)$$

where $s_{p}(t)$ represents the discretized baseband waveform corresponding to the *p*-th signal with the direction $\theta_{p}$ and n(t) is the independent and identically distributed complex white Gaussian noise with zero mean and variance $\sigma_{n}^{2}I$, where **I** is the (N+2M−1)×(N+2M−1) identity matrix. $a(\theta_{p})$ is the steering vector corresponding to the *p*-th signal with the direction $\theta_{p}$, which is defined as:

(5) $$a(\theta_{p})={\left[e^{-j(2\pi /\lambda )k_{1}\sin\theta_{p}},\cdots ,e^{-j(2\pi /\lambda )k_{N+2M-1}\sin\theta_{p}}\right]}^{T}$$

The theoretical covariance matrix of the coprime array snapshot vector x(t) is expressed as:

(6) $$R=E\{x(t)x^{H}(t)\}=\sum_{p=1}^{P}\sigma_{p}^{2}a(\theta_{p})a^{H}(\theta_{p})+\sigma_{n}^{2}I$$

where E{⋅} is the statistical expectation operator, ${\left(\cdot \right)}^{H}$ is the Hermitian transpose, ${\left\{\sigma_{p}^{2}\right\}}_{p=1}^{P}$ are the powers of the impinging signals. In practice, the theoretical covariance matrix **R** cannot be available, which is approximately computed as:

(7) $$\hat{R}=\frac{1}{T}\sum_{t=1}^{T}x(t)x^{H}(t)$$

where $\hat{R}$ is called as the sample covariance matrix and *T* is the number of snapshots. It should be noted that $\hat{R}$ converges to **R** when *T* tends to infinity. If *T* is small, the large mismatch between $\hat{R}$ and **R** can degrade the estimation accuracy.

## 3. Proposed Coprime Array DOA Estimation Method

The theoretical covariance matrix **R** of the coprime array can be vectorized as the vector:

(8) $$z=\mathrm{vec}(R)=Bp+\sigma_{n}^{2}i$$

where vec(⋅) is the vectorization operator which stacks all columns of a matrix on top of the another, $p={\left[\sigma_{1}^{2},\sigma_{2}^{2},\cdots ,\sigma_{P}^{2}\right]}^{T}$, i=vec(I), and the matrix $B\in {\mathbb{C}}^{{\left(N+2M-1\right)}^{2}\times P}$ is given by:

(9) $$B=[a^{*}(\theta_{1})⊗a(\theta_{1}),a^{*}(\theta_{2})⊗a(\theta_{2}),\cdots ,a^{*}(\theta_{P})⊗a(\theta_{P})]$$

where ${\left(\cdot \right)}^{*}$ is the conjugate operation and ⊗ is the Kronecker product operation. It is interesting that **z**, **B**, **p** and **i** in Equation (8) can be respectively regarded as the received signal, array manifold, waveform vector, and noise components of an augmented virtual array. The entries of $a^{*}(\theta_{p})⊗a(\theta_{p})$ in matrix **B** can be denoted as $e^{-j(2\pi /\lambda )(k_{m}-k_{n})\sin\theta_{p}}$, m,n=1,2,⋯,N+2M−1. In the augmented virtual array, the virtual sensor positions are included in the set:

(10) $$S_{p}=\{k_{m}-k_{n},m,n=1,2,\cdots ,N+2M-1\}$$

The difference coarray of the coprime array is defined as:

(11) $$S_{c}=\{\pm (Mn-Nm)d,0\leq m\leq 2M-1,0\leq n\leq N-1\}$$

It has been shown in [35] that a virtual ULA with continuous sensors ranging from (−*MN* − *M* + 1)*d* to (*MN* + *M* − 1)*d* can be extracted from the augmented virtual array. The positions of the virtual sensors in the virtual ULA are contained in the set:

(12) $$S_{v}=\left\{(-Q+1)d,\cdots ,0,\cdots ,(Q-1)d\right\}$$

where *Q* is given by *Q* = *MN* + *M.* By removing the repeated rows of **z** and arranging the rest of rows corresponding to the elements in $S_{v}$, the observation vector of the virtual ULA can be expressed as

(13) $$z_{v}=B_{v}p+\sigma_{n}^{2}i_{v}$$

where $B_{v}\in {\mathbb{C}}^{(2Q-1)\times P}$ is the manifold matrix of the virtual ULA whose elements can be denoted as $e^{-j(2\pi /\lambda )x\sin\theta }$, $x\in S_{v}$, θ∈Θ. $i_{v}$ consists of zeros except a 1 at the *Q-*th position. Based on the observation vector of the virtual ULA $z_{v}$, the covariance matrix of the observation vector $z_{v}$ can be written as:

(14) $$R_{v}=z_{v}z_{v}^{H}$$

Since the observation vector $z_{v}$ behaves like single snapshot in the virtual ULA, the rank of the covariance matrix $R_{v}$ is one. Hence, the traditional subspace methods cannot be implemented on the covariance matrix $R_{v}$ for DOA estimation. To address this problem, the spatial smoothing technique is used to build a positive semidefinite matrix with full rank in [35], which can be performed for DOA estimation. Firstly, the virtual ULA can be divided into *Q* overlapping subarrays with *Q* sensors. Then by using the (*Q* + 1 − *l*)-th to (2*Q* − l)-th rows of $z_{v}$, the received signal vector of the *l-*th subarray is given by:

(15) $$z_{vl}=B_{vl}p+\sigma_{n}^{2}i_{vl}$$

where $B_{vl}\in {\mathbb{C}}^{Q\times P}$ contains the (*Q* + 1 − *l*)-th to (2*Q* − l)-th rows of $B_{v}$, and $i_{vl}$ contains zeros except for a 1 at the *l-*th position. By taking the average of the covariance matrix over all overlapping subarrays, the spatially smoothed matrix can be calculated as:

(16) $$R_{s}=\frac{1}{Q}\sum_{l=1}^{Q}z_{vl}z_{vl}^{H}$$

Since $R_{s}\in {\mathbb{C}}^{Q\times Q}$ is a full-rank matrix, the MUSIC algorithm can be directly performed on $R_{s}$ for DOA estimation. However, because of the exploitation of the spatial smoothing technique, the computational complexity is greatly increased, especially when the number of physical sensors is very large. Besides, the achieved aperture in the virtual ULA can be significantly reduced by the spatial smoothing technique, which results in performance degradation in the DOA estimation. 

Instead of the spatial smoothing technique, we propose a highly accurate and computationally efficient method to reconstruct a full-rank Toeplitz covariance matrix. For the virtual ULA, the element of the observation vector $z_{v}$ is denoted as:

(17) $$z_{va}=\sum_{p=1}^{P}\sigma_{p}^{2}e^{-j(2\pi /\lambda )da\sin\theta_{p}}+\sigma_{n}^{2}i_{a}$$

where a=(−Q+1),⋯,0,⋯,(Q−1) and $i_{a}$ is given by:

(18) $$i_{a}=\left\{ \begin{array}{l} 1,a=0\\ 0,a\neq 0 \end{array}\right.$$

Based on the elements of the observation vector $z_{v}$, the element of the covariance matrix $R_{v}$ can be expressed as:

(19) $$\begin{array}{ll} r(a,b) & =E\{z_{va}z_{vb}^{*}\}\\ & =\left[\sum_{p=1}^{P}\sigma_{p}^{2}e^{-j(2\pi /\lambda )da\sin\theta_{p}}+\sigma_{n}^{2}i_{a}\right]\times {\left[\sum_{l=1}^{P}\sigma_{l}^{2}e^{-j(2\pi /\lambda )db\sin\theta_{l}}+\sigma_{n}^{2}i_{b}\right]}^{*}\\ & =\sum_{p=1}^{P}\sigma_{p}^{2}e^{-j(2\pi /\lambda )da\sin\theta_{p}}\sum_{l=1}^{P}\sigma_{l}^{2}e^{j(2\pi /\lambda )db\sin\theta_{l}}+\sigma_{n}^{2}i_{b}\sum_{p=1}^{P}\sigma_{p}^{2}e^{-j(2\pi /\lambda )da\sin\theta_{p}}+\sigma_{n}^{2}i_{a}\sum_{l=1}^{P}\sigma_{l}^{2}e^{j(2\pi /\lambda )db\sin\theta_{l}}+\sigma_{n}^{4}\delta_{a,b}\\ & =\sum_{l=1}^{P}\sigma_{l}^{2}e^{j(2\pi /\lambda )db\sin\theta_{l}}\left[\sum_{p=1}^{P}\sigma_{p}^{2}e^{-j(2\pi /\lambda )da\sin\theta_{p}}+\sigma_{n}^{2}i_{a}\right]+\sigma_{n}^{2}i_{b}\sum_{p=1}^{P}\sigma_{p}^{2}e^{-j(2\pi /\lambda )da\sin\theta_{p}}+\sigma_{n}^{4}\delta_{a,b} \end{array}$$

where a,b=(−Q+1),⋯,0,⋯,(Q−1). Let us define:

(20) $$c_{a,l}=\sigma_{l}^{2}\left[\sum_{p=1}^{P}\sigma_{p}^{2}e^{-j(2\pi /\lambda )da\sin\theta_{p}}+\sigma_{n}^{2}i_{a}\right]$$

and

(21) $$g_{a,b}=\sigma_{n}^{2}i_{b}\sum_{p=1}^{P}\sigma_{p}^{2}e^{-j(2\pi /\lambda )da\sin\theta_{p}}$$

Therefore, r(a,b) in Equation (19) can be further written as:

(22) $$r(a,b)=\sum_{l=1}^{P}c_{a,l}e^{j(2\pi /\lambda )db\sin\theta_{l}}+g_{a,b}+\sigma_{n}^{4}\delta_{a,b}$$

Here, $g_{a,b}$ can be further computed as:

(23) $$g_{a,b}=\left\{ \begin{array}{l} h_{a},b=0\\ 0,\;b\neq 0 \end{array}\right.$$

with

(24) $$h_{a}=\sigma_{n}^{2}\sum_{p=1}^{P}\sigma_{p}^{2}e^{-j(2\pi /\lambda )da\sin\theta_{p}}$$

By using any non-zero row of the covariance matrix $R_{v}$, a Toeplitz covariance matrix can be reconstructed as following:

(25) $$\begin{array}{l} R_{t}(a)=\left[\begin{array}{cccc} r(a,0) & r(a,1) & \cdots & r(a,Q-1)\\ r(a,-1) & r(a,0) & \cdots & r(a,Q-2)\\ \vdots & \vdots & \ddots & \vdots \\ r(a,-Q+1) & r(a,-Q+2) & \cdots & r(a,0) \end{array}\right]\\ \;=A_{t}\mathrm{diag}\{c_{a,1},\cdots ,c_{a,P}\}A_{t}^{H}+h_{a}I_{Q}+\sigma_{n}^{4}I_{Q,a} \end{array}$$

where $I_{Q}$ is the Q×Q identity matrix and the matrix $A_{t}$ is given by

(26) $$A_{t}=\left[a_{t}(\theta_{1}),a_{t}(\theta_{2}),\cdots ,a_{t}(\theta_{P})\right]$$

with

(27) $$a_{t}(\theta_{p})={\left[1,e^{-j(2\pi /\lambda )d\sin\theta_{p}},\cdots ,e^{-j(2\pi /\lambda )d(Q-1)\sin\theta_{p}}\right]}^{T}$$

The matrix $I_{Q,a}$ is denoted as:

(28) $$I_{Q,a}=\left\{ \begin{array}{l} I_{Q},a=0\\ 0_{Q},a\neq 0 \end{array}\right.$$

where $0_{Q}$ is a Q×Q zero matrix. From Equation (20), it can be observed that $c_{a,l}\neq 0$ for l=1,⋯,P. Besides, the matrix $A_{t}$ is a Vandermonder matrix according to its definition in (26). Therefore, the Toeplitz covariance matrix $R_{t}(a)$ reconstructed in (25) is full-rank, which can offer a large enough rank to detect the directions of *P* sources. 

Subsequently, we implement the ESPRIT algorithm on the reconstructed Toeplitz covariance matrix $R_{t}(a)$ to estimate the directions of sources. An eigendecomposition of the Toeplitz covariance matrix $R_{t}(a)$ can be performed as follows:

(29) $$R_{t}(a)=U_{s}\Lambda_{s}U_{s}^{H}+U_{n}\Lambda_{n}U_{n}^{H}$$

where $\Lambda_{s}$ contains the *P* largest eigenvalues of the Toeplitz covariance matrix $R_{t}(a)$, and its corresponding eigenvectors are contained in the signal subspace matrix $U_{s}$. The remaining eigenvalues and corresponding eigenvectors of the Toeplitz covariance matrix $R_{t}(a)$ are contained in the diagonal matrix $\Lambda_{n}$ and the noise subspace matrix $U_{n}$, respectively. 

The Vandermonder matrix $A_{t}$ can be partitioned as follows:

(30) $$A_{t}=\left[\begin{array}{c} A_{t1}\\ A_{t}(Q,:) \end{array}\right]=\left[\begin{array}{c} A_{t}(1,:)\\ A_{t2} \end{array}\right]$$

where $A_{t1}$ comprises of the first *Q* − 1 rows of the matrix $A_{t}$ and $A_{t2}$ comprises of the last *Q* − 1 rows of the matrix $A_{t}$. According to the definition of the Vandermonder matrix $A_{t}$ in (26), there exists a rotation matrix Γ satisfying:

(31) $$A_{t2}=A_{t1}\Gamma$$

where the rotation matrix Γ is defined as

(32) $$\Gamma =\mathrm{diag}\{e^{-j(2\pi /\lambda )d\sin\theta_{1}},e^{-j(2\pi /\lambda )d\sin\theta_{2}},\cdots ,e^{-j(2\pi /\lambda )d\sin\theta_{P}}\}$$

Similarly, the signal subspace matrix $U_{s}$ can be partitioned as:

(33) $$U_{s}=\left[\begin{array}{c} U_{s1}\\ U_{s}(Q,:) \end{array}\right]=\left[\begin{array}{c} U_{s}(1,:)\\ U_{s2} \end{array}\right]$$

where $U_{s1}$ represents the first *Q* − 1 rows of the signal subspace matrix $U_{s}$ and $U_{s2}$ represents the last *Q* − 1 rows of the signal subspace matrix $U_{s}$. Because the columns of the matrix $A_{t1}$ and the columns of the matrix $U_{s1}$ span the identical subspace, there exists a nonsingular matrix **F** satisfying the following equations:

(34) $$U_{s1}=A_{t1}F$$

and

(35) $$U_{s2}=A_{t2}F=A_{t1}\Gamma F$$

Based on Equations (34) and (35), we can obtain the following relationship:

(36) $$F^{-1}\Gamma F=U_{s1}^{+}A_{t1}\Gamma F=U_{s1}^{+}U_{s2}$$

where ${\left(\cdot \right)}^{+}$ denotes the Moore-Penrose inverse.

From Equation (36), it can be observed that Γ and $U_{s1}^{+}U_{s2}$ are similar matrices, which should have the same eigenvalues. It should be noted that the eigenvalues of the rotation matrix Γ can be given by $e^{-j(2\pi /\lambda )d\sin\theta_{i}},i=1,2,\cdots ,P$. Therefore, the DOA of the source signals can be estimated from:

(37) $${\hat{\theta }}_{i}=\arcsin\left(-\frac{1}{\pi }\mathrm{angle}(u_{i})\right)i=1,2,\cdots ,P$$

where $u_{i},i=1,2,\cdots ,P$ are the eigenvalues of $U_{s1}^{+}U_{s2}$, and angle(⋅) represents the phase angle of a complex number.

The proposed coprime array DOA estimation method is summarized in Table 1. The related remarks are listed as follows:

**Remark 1.**  *The computational complexity of the proposed coprime array DOA estimation method is*$O({\left(N+2M-1\right)}^{2}T+Q^{3}+3QP^{2}+2P^{3})$*, which is mainly caused by the eigendecomposition of the Toeplitz covariance matrix*$R_{t}(a)$*with a complexity of*$O(Q^{3})$. *The computational complexity of the coprime MUSIC algorithm [35] mainly lies in the peaking search step with*$O(Q^{2}J)$*, where J is the number of hypothetical angles. In general cases,*J≫Q*is used to ensure the satisfactory resolution performance of the DOA estimation. Therefore, the proposed method has lower computational complexity than the coprime MUSIC algorithm [35]. Compared to the sparsity-based DOA estimation method in coprime array [36] with the complexity of*$O({\left(N+2M-1\right)}^{2}J)$, *the proposed method also has lower computational cost and does not encounter the trade-off between the estimation performance and the computational complexity.*

**Remark 2.**  *The proposed coprime array DOA estimation method has the following advantages. Firstly, compared to the traditional DOA estimation methods with ULA structure, the coprime array configuration used in the proposed method can identify more DOAs of sources with limited sensor number due to increased DOFs in the coarray domain. Secondly, different from the spatial smoothing technique, we use the symmetrical property of the virtual uniform linear array to construct a full-rank Toeplitz covariance matrix. In such way, the computational burden can be significantly reduced and the estimation accuracy can be greatly improved. Last but not least, we derive a closed-form expression for efficiently estimating the DOAs of sources, which circumvents the use of predefined spatial angular grids and the peaking search step that are employed in traditional coprime MUSIC and sparsity-based methods. Based on the above advantages, the solutions of the proposed method can be used for equipment working in real conditions due to fast and precise calculation.*

## 4. Simulation Results

In this section, a coprime array with *M* = 3 and *N* = 5 is deployed. In this deployed coprime array, we assume that the first sensor of two subarrays is collocated at the zeroth position for reference. Therefore, it is obvious that the deployed coprime array has *N* + 2*M* − 1 = 10 physical sensors in total, whose positions are included at $k={\left[0,3d,5d,6d,9d,10d,12d,15d,20d,25d\right]}^{T}$. The unit inter-element spacing *d* is set to be half of wavelength. 

We compare the performance of the proposed coprime array DOA estimation method with the partial spectral search method [32], the coprime MUSIC method [35], and the sparsity-based method [36]. For the partial spectral search method, coprime MUSIC method and sparsity-based method, the hypothetical angular grids are within $\left[-90^{\circ },90^{\circ }\right]$ with the fixed angular step of $0.1^{\circ }$. For the sparsity-based method, the regularization variable is set as 0.25 as recommended in [36]. Throughout the simulations, the sensor noise is assumed to be complex white Gaussian noise with zero mean and unit variance.

**Figure 2:**
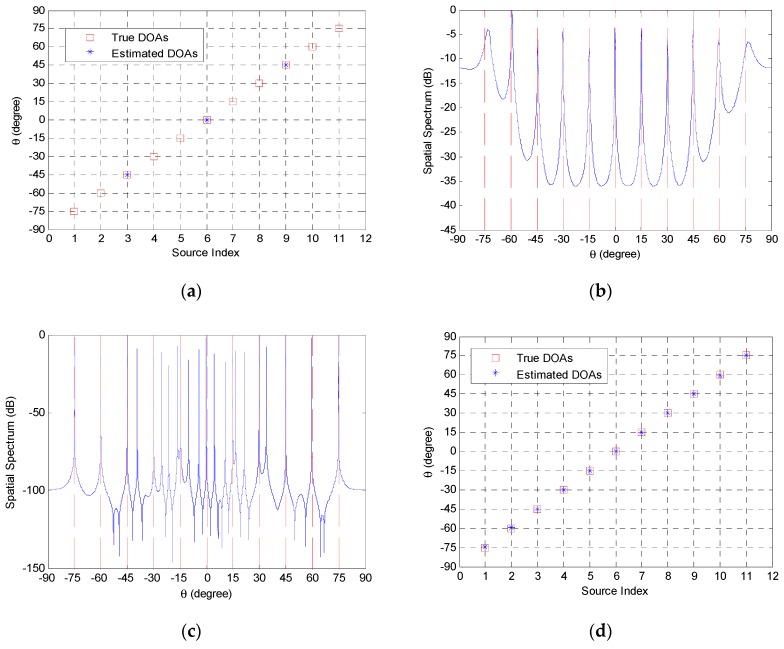
The resolution performance of each method with coprime array when the number of sources is larger than that of physical sensors: (**a**) the partial spectral search method; (**b**) the coprime MUSIC method; (**c**) the sparsity-based method; (**d**) the proposed method; first example.

**Figure 3:**
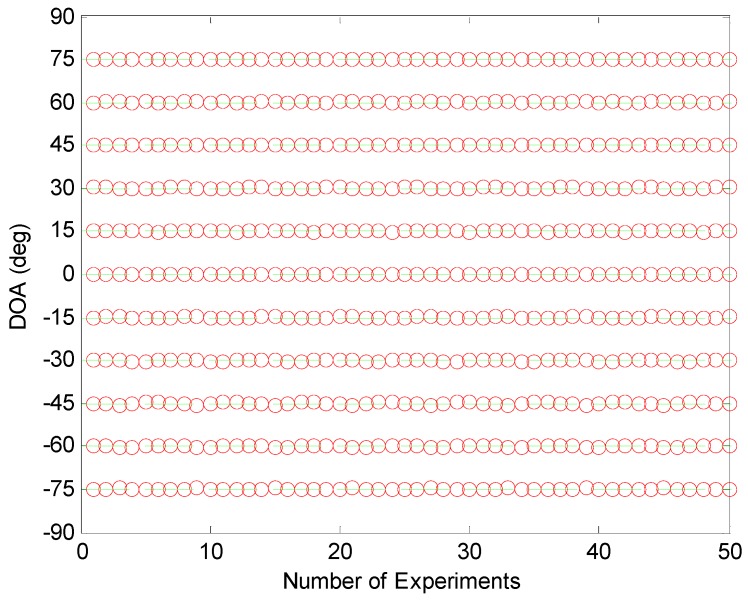
Resolution ability test of the proposed method in different trials; first example.

In the first example, the resolution ability of the aforementioned methods is examined when the number of sources is larger than that of physical sensors. Assume that 11 uncorrelated sources with equal power impinge on the coprime array from $-75^{\circ }$, $-60^{\circ }$, $-45^{\circ }$, $-30^{\circ }$, $-15^{\circ }$, $0^{\circ }$, $15^{\circ }$, $30^{\circ }$, $45^{\circ }$, $60^{\circ }$, $75^{\circ }$. The signal-to-noise ratio (SNR) is equal to 10 dB and the number of snapshots in the coprime array is set as *T* = 300. Although the partial spectral search method calculates the spatial spectra of two linear subarrays, it does not compute the spatial spectrum of the entire coprime array. Hence, we plot the estimated DOAs of the partial spectral search method in the entire coprime array in Figure 2a. Because the increased DOFs in the coprime array are not used, the partial spectral search method cannot identify the 11 sources completely. We display the spatial spectra of the coprime MUSIC method and the sparsity-based method in Figure 2b,c, respectively. The coprime MUSIC method can detect all of the sources because the increased DOFs in the coprime coarray are utilized. There are several spurious peaks in the spatial spectrum of the sparsity-based method due to the exploitation of the uncertain regularization variable. For the proposed method, the estimated DOAs versus the source index are shown in Figure 2d. It can be observed that the proposed method can handle all of the sources accurately, because the number of available DOFs in the coprime array with *N* + 2*M* − 1 = 10 physical sensors is extended to MN+M−1=17. In order to verify the resolution performance of the proposed method in different trials, Figure 3 presents the estimated DOAs of the above 11 uncorrelated sources using the proposed method in 50 times Monte-Carlo run. The circles denote the estimated DOAs, while the dashed lines denote the actual directions. It can be seen that the proposed method is effective in different trails. 

**Figure 4:**
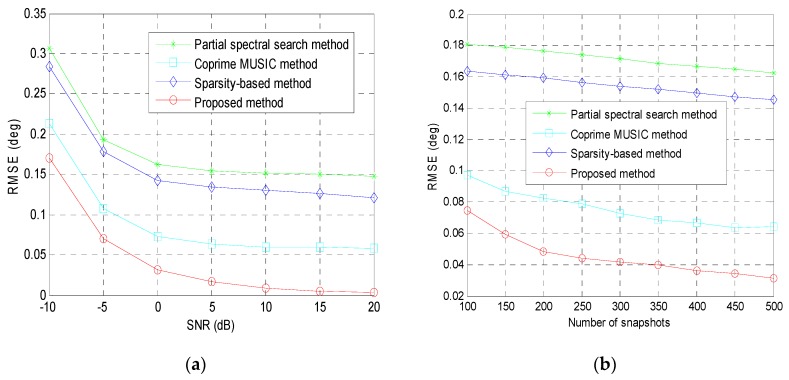
The RMSE performance of all examined methods: (**a**) RMSE versus the input SNR; (**b**) RMSE versus the number of snapshots; second example.

In the second example, we investigate the estimation accuracy of all methods in terms of the root mean square error (RMSE) criterion. The RMSE is defined as:

(38) $$\mathrm{RMSE}=\sqrt{\frac{1}{VP}\sum_{v=1}^{V}\sum_{p=1}^{P}{\left({\hat{\theta }}_{p,v}-\theta_{p}\right)}^{2}}$$

where ${\hat{\theta }}_{p,v}$ is the estimated DOA of the *p*-th source in the *v*-th Monte-Carlo trial, and *V* is the number of Monte-Carlo trials. In this example, 300 rounds of Monte-Carlo trials are conducted. We assume that there is one source randomly generated from the angular interval $\left[0^{\circ },5^{\circ }\right]$. Figure 4a displays the RMSE of the tested methods versus the SNR for fixed training snapshots *T* = 500. It can be observed that the RMSE of the proposed method is smaller than that of the remaining methods, which indicates that the estimation accuracy of the proposed method is better than the remaining methods. The estimation accuracy of the partial spectral search method, coprime MUISC method and sparsity-based method is limited by the hypothetical angular step in the predefined sampling region. On the contrary, the proposed coprime array DOA estimation method does not have such limitations. The RMSE of the aforementioned methods versus the number of snapshots for fixed SNR = 0 dB is shown in Figure 4b. The proposed method still enjoys the best performance, which illustrate that implementing the ESPRIT-like method in the virtual ULA can achieve higher estimation accuracy than the MUSIC-like methods.

In the third example, we compare the computational complexity of all the coprime array DOA estimation methods from the aspect of the estimation time. One signal source is assumed to come from $0^{\circ }$. The SNR and the number of snapshots are set as 10 dB and 500, respectively. 500 Monte-Carlo runs are performed. The running time of each coprime array DOA estimation method is shown in Table 2 based on an Intel Core i5-2450M, 4GB RAM laptop. It is obvious that the estimation time of the proposed method is smaller than that of the other methods, which means that the proposed method has lower computational cost than the other methods. This is because the proposed method avoids the spatial smoothing and peaking search steps. The sparsity-based method has the largest running time and suffers from the heaviest computational burden, since it has to solve the optimization problem and the spectrum search process is exploited.

**Figure 5:**
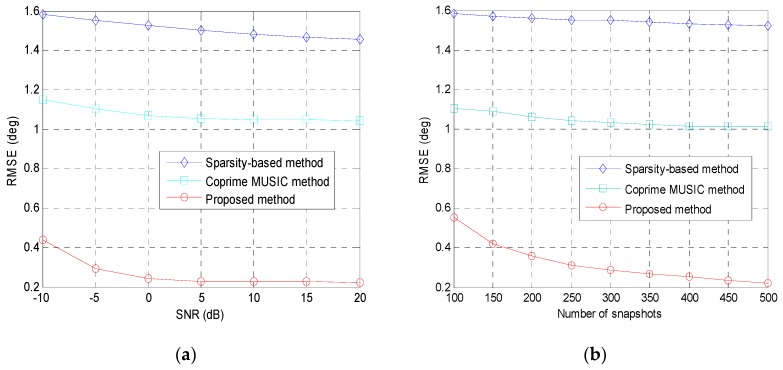
The RMSE performance of the tested methods when there are more sources than the physical sensors: (**a**) RMSE versus the input SNR; (**b**) RMSE versus the number of snapshots; fourth example.

In the fourth example, we test the RMSE performance of different methods when there are more sources than the physical sensors. We assume that 11 sources are incident on the coprime array from the directions uniformly distributed in $\left[-50^{\circ },50^{\circ }\right]$. However, the partial spectral search method cannot detect all of the sources because this method does not use the increased DOFs in the coprime array. Therefore, we only compare our method with the coprime MUSIC method and the sparsity-based method in this example. 500 Monte-Carlo trials will be conducted to obtain each point in the curves of the pictures. The RMSE of the tested methods versus the SNR for fixed training snapshots *T* = 500 is plotted in Figure 5a. It can be observed that the RMSE of the proposed method is smaller than that of the coprime MUSIC method and sparsity-based method, which indicates that the performance of the proposed method is better than the coprime MUSIC method and sparsity-based method. The RMSE of the aforementioned methods versus the number of snapshots for fixed SNR = 10 dB is shown in Figure 5b. As can be seen, the proposed method still outperforms the coprime MUSIC method and the sparsity-based method when there are more sources than the physical sensors.

**Figure 6:**
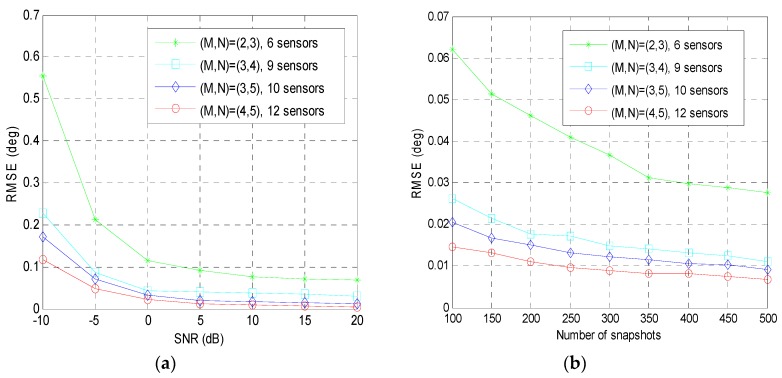
The RMSE performance of the proposed method in different coprime array configurations: (**a**) RMSE versus the input SNR; (**b**) RMSE versus the number of snapshots; fifth example.

In the fifth example, the RMSE performance of the proposed method in different coprime array configurations is taken into consideration. We assume that the coprime pair (*M*,*N*) is respectively set as (2,3), (3,4), (3,5), (4,5) to form four different coprime array geometries and the other variables are chosen as the same as the second example. Figure 6a presents the RMSE of the proposed method versus the SNR for fixed training snapshots *T* = 100, and Figure 6b shows the RMSE of the proposed method versus the number of snapshots for fixed SNR = 10 dB. The RMSE of the proposed method decreases as the input SNR and the number of snapshots increase, which demonstrates that the proposed method can still achieve satisfactory performance in different coprime array configurations.

## 5. Conclusions

We apply the ESPRIT-based method to the symmetrical coprime virtual ULA for DOA estimation. By using the symmetrical structure of the virtual uniform linear array, a Toeplitz covariance matrix can be reconstructed with full rank and the spatial smoothing technique can be circumvented. The ESPRIT method is performed on the reconstructed Toeplitz covariance matrix for DOA estimation without peak searching and predefined angular grids. Finally, a closed-form solution is obtained to resolve the sources. Since the DOFs are increased in the coprime coarray domain, the proposed method can identify more DOAs than the number of physical sensors. The proposed method has improved performance and reduced computational complexity compared to the traditional coprime array DOA estimation methods. Computer simulations demonstrate the advantages of the proposed method over other methods.

## Figures and Tables

**Table 1:** The proposed coprime array DOA estimation method.

**Step 1:** Calculate the observation vector $z_{v}$ of the virtual ULA in (13).
**Step 2:** Compute the covariance matrix $R_{v}$ of the virtual ULA in (14).
**Step 3:** Reconstruct the Toeplitz covariance matrix $R_{t}(a)$ in (25).
**Step 4:** Perform eigendecomposition on $R_{t}(a)$ to obtain the matrix $U_{s}$ in (29).
**Step 5:** Estimate the directions of the sources from (37).

**Table 2:** Running time of each coprime array DOA estimation method.

	Proposed Method	Partial Spectral Search	Coprime MUSIC Method	Sparsity-Based Method
500 runs	0.943 s	44.668 s	34.511 s	1567.693 s
average time	0.002 s	0.089 s	0.069 s	3.135 s

## Abbreviations

The following abbreviations are used in this paper:

DOA	Direction-of-Arrival
ESPRIT	Estimation of Signal Parameters via Rotational Invariance Techniques
MUSIC	MUltiple SIgnal Classification
ULA	Uniform Linear Array
DOF	Degrees-of-freedom
SNR	Signal-to-Noise Ratio
RMSE	Root Mean Square Error
